# 7.B. Workshop: Labor migration, the food supply chain and the COVID-19 syndemic: Germany, Netherlands, and the USA

**DOI:** 10.1093/eurpub/ckac129.404

**Published:** 2022-10-25

**Authors:** Organised by: Bielefeld University (Germany), Chair persons: Nora Gottlieb (Germany), Seth M Holmes (USA)

## Abstract

The agriculture and meat-processing sectors employ, largely, (im)migrant workers in Germany, the Netherlands, and the USA. Deemed “critical infrastructure,” workers in the food supply chain (FSC) were particularly vulnerable to SARS-CoV-2 infection due to poor adaptation of policies and practices related to health, immigration, and work. The pandemic has shone a light on preexisting inequalities and risks related to precarious and hazardous work, particularly for (im)migrant workers. We conducted harmonized policy analyses in North-Rhine Westphalia, Germany, in the Netherlands, and in Illinois, USA on measures taken during the pandemic and how they affected migrant workers. These three regions host large businesses in meat-processing and agriculture. They saw significant COVID-19 outbreaks, with widespread health, social and economic repercussions. Public health effects included higher risk for all communities in the region; social effects included local lockdown measures; economic effects included food supply problems, euthanized animals, and financial losses due to the temporary closure of businesses. In our workshop, we will deliver three brief presentations on (im)migration, labor, and occupational safety and health policies during the COVID-19 syndemic in Germany, the Netherlands, and the USA. We present results of our analysis, highlighting similarities and differences between the EU-member states Germany and the Netherlands, on the one hand, and the US, related to the employment and exploitation of migrant workers from the global South/East in the global North/West. We highlight issues to be addressed in future global emergencies. The pandemic has prompted occupational safety and health measures in the FSC. However, agro-industrial sectors in Western Europe and the US heavily rely on precariously employed workers (e.g., seasonal, temporary and/or subcontracted workers). This may impact the extent to which preventive measures “work” for these populations: even when good policies are formulated, information exchange, collaboration, and enforcement by relevant agencies and immigration authorities is critical. Without such cooperation, the realization of stronger social protection of (im)migrant workers is are left to the good will of the employer. Structural drivers in the form of policies related to mobile labor, (im)migration and employment, superimposed on segmented labor markets and discriminatory practices, drove significant morbidity among (im)migrant workers. The following discussion will synthesize insights from the three contexts, and provide an opportunity to brainstorm with workshop participants to facilitate cross-national collaboration on this issue.

**Key messages:**

• Food supply chain workers are at the intersection of discrimination in health, employment, and migration.

• Comparative analysis provides a rich framework to guide management of vulnerable sectors, workers and workplaces during future pandemics.

